# The Medical Aspect of Trephining in Epilepsy

**Published:** 1895-10

**Authors:** Eugene Foster

**Affiliations:** Augusta, Ga., Professor of Principles and Practice of Medicine, and State Medicine, Medical Deparment University of Georgia


ATLANTA

### Medical and Surgical Journal.

VOL. XII. OCTOBER, 1895. NO. 8.

LUTHER B. GRANDY, M.D., MANAGING EDITOR.

M. B. HUTCHINS, M.D., BUSINESS MANAGER.

COLLABORATORS:
H. V. M. MILLER, M.D., LL.D., VIRGIL O. HARDON, M.D., FLOYD W. McRAE, M.D., DUNBAR ROY, M.D., AND H. R. SLACK, M.D., PH.M.

### *ORIGINAL COMMUNICATIONS.*

### THE MEDICAL ASPECT OF TREPHINING IN EPILEPSY.*

BY EUGENE FOSTER, M.D., AUGUSTA, GA.,

Professor of Principles and Practice of Medicine, and State Medicine, Medical Deparment University of Georgia.

MR. PRESIDENT: Your program committee very kindly requested me to submit at this meeting a paper on "The Medical Aspect of Trephining in Epilepsy."

I take it, Mr. President, that I am expected to say if or not trephining is necessary or justifiable in the treatment of epilepsy. So understanding my duty, I shall shoot at this mark.

The answer to this question can be made only after an intelligent conception of what epilepsy really is.

What is epilepsy? Epilepsy proper—*i. e.*, "genuine epilepsy," "idiopathic epilepsy," is a chronic functional neurosis; *i. e.*, it is a malady in which, so far as ascertainable by all the methods of in-

Read before the Augusta Academy of Medicine, August 12, 1895.

vestigation known to modern medicine, there is no constant lesion of the nervous system. In other words, epilepsy is a malady devoid of pathological anatomy. Therefore, it is a purely functional disease. This being so, Mr. President, it seems to me that trephining has no legitimate position in the treatment of this malady.

Not only has epilepsy, the idiopathic variety, no known morbid anatomy, but we are forced to confess that as to the cause of the affection we know absolutely nothing. Neither can we say whether it is a disease or merely a symptom of disease. Now, sir, I ask if it is proper, is it justifiable to trephine the skull in the vain hope of curing or even mitigating a malady who sepathological anatomy is a myth, and whose causation is utterly unknown to us? What is the rational indication for trephining a case of idiopathic epilepsy? Absolutely none on earth. Now I am aware that there are a few enthusiasts who will insist that in this day of antiseptic and aseptic surgery, in this day of brilliant brain surgery, trephining the skull and excising brain tissue is a perfectly safe operation. That this being so, the surgeon is justified in boldly laying open the cranium, probing into the brain and searching for the cause of this deplorable malady. Indeed, it seems that some few would regard it as criminal not to make this exploration. Now, sir, I have as much admiration for the achievements of modern medicine as you or any member of this Academy. In common with the profession generally I have observed with astonishment the boldness of modern brain surgery. Occasionally operations having been performed successfuly, which twenty years ago would have been considered necessarily fatal. We read of surgeons boldly trephining the cranium, and, in some instances, leaving but little of the skull to the patient who had previously been taught by school hygiene and physiology to regard it as a most necessary part of his bodily make-up. We have arrived upon the day when we complacently accept the advice of Wyeth to make a "trap-door" in the skull in order that large areas of the brain may be explored. We behold surgeons, after making this trap-door arrangement in the skull, cut through the membranes and complacently push a probe through various parts of a fellow's head—the justification for this, to me, daring process being that the cortex cerebri is apparently sound when it was thought to be diseased, and that there must be an abscess or a cyst in the deeper structures, as evidenced by the patient having a paralysis, epilepsy, or psychical disturbance. Not content with this fierce onslaught upon the very citadel of life, they go further, and cut and slash out parts of the brain as if it were an ordinary muscle of the body. Some of these operations are truly marvelous in their conception and execution. Some few of them are successful. The successful ones are printed in flaming head-lines, but very few failures ever go before the profession. But, Mr. President, with rare exceptions, these cases are mere surgical novelties —in the vast majority of cases valuable rather as proof of how much rough usage the brain can stand than for any real benefits which have resulted to suffering humanity. Now, sir, I insist that because modern brain surgery, so-called, has developed the fact that the brain may be incised, even large portions of it removed, with occasionally apparent benefit to the individual operated upon, and in numerous other instances attended with no alarming results, this is no justification for the claim that such operations are perfectly safe, and, therefore, that we are warranted in making exploratory operations upon the brain as is the rule to do exploratory laparotomies. Here and now I enter my protest against considering exploratory brain surgery as analogous to exploratory laparotomy. Would that it were true, but unfortunately for the surgeon, and more so to the patient, the complicated structure of the brain renders cerebral diseases far more serious and far less amenable to cure by surgical procedure than in lesions of the abdominal viscera. I grant that modern surgery has demonstrated the fact that operations upon the cranium and brain are safer than they were once thought to be, but I insist that notwithstanding the diminished dangers of modern aseptic and antiseptic methods, exposing the brain and probing into it and cutting out any portion of it is a capital operation, and should never be undertaken except after mature deliberation, and only as a *dernier ressort*. Almost all of our modern text-books plant themselves squarely upon the proposition that idiopathic epilepsy is unsuited to operative treatment. No less an authority than "The American Text-book of Surgery" takes this position, and warns the profession against the new-born boldness begotten of success in cerebral surgery. Let us heed this warning.

If there be any of you who deem me an alarmist in regarding this a capital operation, I remind you that "The American Text-book of Surgery," page 528, records the fact that in fifteen operations for supposed intercranial tumors, but in which no tumor was found at the seat of the operation, thirteen died. Thirteen deaths out of fifteen cases so operated upon. The mortality is simply appalling. What caused it? Unwarranted interference, meddlesome surgery. The cases had been diagnosticated as cerebral tumors, yet they found the cortex cerebri sound. But the diagnosis of cerebral tumor had been made in each of these cases, so the operators felt that as the tumor was not located on the surface of the brain, it was nevertheless near by. So they probed and punched through the brain of these fifteen unfortunates—the result being a failure to find a tumor in any one instance, but they punched the life out of thirteen of these victims of meddlesome surgery. Again, it is proven to be a grave operation by the mortality in the hands of our home surgeons. It is unnecessary to cite the instances, yet it is a well-known fact that within the last few years several of our citizens have been consigned to the shades of the silent city, the result of trephining the cranium. The well-known, the unquestionable skill of the operators in whose hands these fatalities occurred is a guarantee that all the skill known to the most advanced brain surgery of this day was exhausted in the performance of these operations, and yet the fact that the daisies keep silent watch over the mortal remains of these victims of trephining, clearly to me demonstrates that the operation is a dangerous one. If the operation is occasionally so disastrous in the hands of experts, what would it be in the hands of surgical tyros? So much for idiopathic epilepsy.

Symptomatic epilepsy, on the other hand, may, and not infrequently does, seem to call for operative treatment. The difference between idiopathic and symptomatic epilepsy is as different as day and night. Why? The idiopathic variety occurs wholly independent of cerebral lesions. The symptomatic variety is found associated with gross organic changes in the brain or its coverings—

*i. e.*, in all cases originating primarily in the brain. You are, of course, aware that the symptomatic variety not infrequently arises from irritations located in parts of the body other than the brain. Now, Mr. President, while many cases of symptomatic epilepsy seem to demand operative treatment, I must insist that even here operative interference by trephining, and, if need be, excision of parts of the brain or its coverings, should be undertaken only after the closest study of each case, and in the exercise of a discriminating judgment on the part of the surgeon. Routine practice has no rational place in either the medical and surgical treatment of epilepsy, and especially is this true of trephining.

Here is an instructive instance enforcing this rule. Dr. Briggs reports the case of an epileptic girl who had an old, depressed fracture of the skull, and also necrosis of the tibia. Certainly, by all the rules laid down by the most cautious and expert brain surgery of to-day the inference was fair, indeed the proof well-nigh positive, that the epilepsy was caused by the old depressed fracture of the skull, and any surgeon would have been fully authorized by all rules of modern surgery to have trephined this patient for relief of her deplorable malady. Yet he would have made a mistake. Briggs, being a medical philosopher, and knowing that irritations in other parts of the body than the brain occasionally caused epilepsy, determined to first operate upon the tibia, and if this did not produce a cure of the malady then he would trephine the skull. He was gratified to find that the operation upon the necrosed tibia effected a cure of the epilepsy. The cure was complete, the patient having had no return of the malady during the five subsequent years in which she was kept under observation. I grant that this was quite an exceptional case, but it certainly impresses upon us the lesson of great caution even in cases in which *prima facie* evidence points to depressed cranial fractures as the cause of epilepsy. Unquestionably injuries of the head which have caused a fracture of the skull, or lesions of the brain or its coverings, are not infrequently traumatic irritants. In all cases wherein epilepsy can be traced directly to an injury of the head, the question of the necessity or propriety of trephining forces itself upon the intelligent physician. If there be evidence of old or recent fracture of the cranium, or of a lesion of the brain or its coverings, the operation of trephining is justifiable, provided all other irritants known to be capable of producing epilepsy have been diligently sought for and removed and yet the convulsions recur. In some instances epilepsy is said to arise from a trauma of the head, yet the skull may not be depressed, nor the brain or its coverings injured, the only discoverable lesion being the scar of the scalp. In numerous instances operators have reported cure of epilepsy by the mere excision of the cicatrix in the scalp. In such cases the cicatrix should be thoroughly removed, and if subsequent experience demonstrates the necessity for trephining it should be performed, and if either the dura or the brain be the seat of the lesion, excision of the offending part or parts may in some rare instances be made a part of the trephining operation. But suppose after removing the depressed portion of the skull the dura seems healthy, what then? Shall the operation cease with the mere removal of the depressed bone? "The American Text-book of Surgery" lays down this rule: "After trephining the skull, the dura should always be opened, even though it be sound, in order to inspect the brain." I do not assent to this rule. Incision of the dura adds to the gravity of an already serious operation. In such cases the operation should stop with that of trephining, and if the epilepsy continues thereafter then, by a secondary operation, if necessary, the dura may be incised and the brain explored.

In symptomatic epilepsy consequent on head injuries, we should remember that the epilepsy may not, and, in fact, does not in the vast majority of cases, make its appearance until months, or even years, after the trauma has been received. This being a fact, it is to me exceedingly strange that the brain quietly submits to these irritants for such great lengths of time. To me the fact seems to indicate that something has been superadded to the trauma. In other words, such cases seem to indicate that the epilepsy would have occurred in those individuals had there never been a head injury. If I am asked to explain why trephining cures these cases, I answer, first, that trephining, even with excision, in the vast majority of cases signally fails to cure them. This is a common experience of the profession. Second, no fact is better established in medical science than that accidental injuries, or surgical operations upon other parts than the head, have in countless instances either markedly mitigated the severity or actually cured cases of epilepsy in individuals having head lesions. Almost every one of our text-books cite the fact that epilepsy has been known to disappear after such patients had accidentally been burned by falling in a fire, or had a limb amputated.

Again, I would insist that even in epilepsy in individuals the subjects of head injuries, trephining is only an experiment. No surgeon can promise a cure in such cases, no matter if the trephining be done for the removal of either recent or old head lesions; indeed, he cannot even promise that his efforts to cure them will not add further damage. I would not be misunderstood just here. Trephining is justifiable in such cases, but that the operation is necessary is abundantly disproven by its failures to cure the vast majority of such patients. Trephining such cases is necessary only to insure removal of the possible cause of the persistence of the epilepsy.

What of Jacksonian epilepsy?

Before we can answer this question we must understand what is meant when we speak of Jacksonian epilepsy. This term is applied to those cases in which there is spasm of a limited group or groups of muscles, usually unattended by loss of consciousness, and in which therefore the discharging lesion seems clearly to be limited to the center or centers corresponding to the muscles involved. When the spasm commences locally—in one limb, for instance, it would indicate local excessive instability of the corresponding cortical center, and not infrequently established organic disease. Then comes the question, shall the operation of trephining be done and the disturbing center be removed? The answer depends, in my judgment, on the condition of the discharging center. If there be a gross, macroscopic local lesion of the center, it may be justifiable to remove it. Suppose, however, there be no lesion of the center corresponding to the spasm. What then? Shall the center be excised? Numerous operators have insisted that even in such cases as the latter the center should be removed, and they have put their theory into practice. They determined the location of the center by electric stimulation, and then cut out the whole area. What was the result? In a small fraction of cases cures are said to have resulted; in another small fraction the epilepsy was said to have been mitigated either as to intensity or frequency of attacks, while in the vast majority of instances the operation failed of benefiting the individuals, and in some instances added to the existing damage. "The American Text-book of Surgery" says of operative treatment of Jacksonian epilepsy: "The time has not yet come when a definite opinion can be expressed as to the value of these operations. The danger to life is not very great. A large number of the cases have not been bettered, but in many great relief has followed both in diminished severity and diminished frequency of the attacks." Under all these facts, Mr. President, while operative treatment of Jacksonian epilepsy may be justifiable in selected cases, as a general proposition it is to be condemned in the present state of uncertainty of results.

### FOCAL EPILEPSY.

Occasionally this variety is encountered. In a given number of cases in which there has been no lesion of which there was evidence by scar, depressed bone or other signs, in which there was unconsciousness in association with general epileptic spasm instead of monospasm—*i. e.*, spasm of an arm or leg, but the attacks always commenced in a certain set of muscles as a focus, from which they radiated to other centers, the term focal epilepsy has been applied. In numerous such instances attempts have been made to either cure the disease or prevent the extension of the epileptic convulsions to other centers, by removing the center or centers corresponding to the muscles in which the convulsions commenced. Now for results. Up to date only a small fraction of cases have been reported as cured; all of them, however, have been reported before sufficient time has elapsed to enter up an impartial judgment; in a large per cent. reporters report improvement as manifested by decreased severity or frequency, but in the vast majority of cases no improvement followed these operations. Only one important decision can be made to justify the operation—*i. e.*, that it has not as a rule seemed to increase the frequency or number of attacks of epilepsy. The number of reported deaths following the operation is comparatively small. But mark you, reported cases. Operators are not fond of reporting deaths following operations, and this, in my opinion, accounts for the small list of published fatalities in this field. Not only because of its numerous failures, but from the reasons below given, I do not believe the operation a justifiable one, except in a few selected cases. Dowse asks, "Is the operation justifiable?" He answers: "If the fits generally spread through the whole of one side, and still more if they often spread to the other side, the probability of benefit is too small to justify the very considerable risk of the operation. Apparently the repeated discharges lead to so wide a deficiency in the stability of the nerve cells that the discharge starts from other regions if the primary lesion is removed. Hence, the operation should never be thought of in a case of idiopathic epilepsy in which the fits begin locally, because it is certain that the discharging tendency is widespread and almost certainly adequate, in other parts, to cause fits."

"The American Text-book of Surgery" says of focal epilepsy: "In these cases, even more possibly than of the Jacksonian type, it is necessary to have reliable and repeated observations of the attacks before deciding to operate. Even with the diminished dangers of modern cerebral surgery, exposing the brain, and especially excising a cortical center, is not a trifling operation, but a serious one, which must not be undertaken without the best evidence of its necessity."

Now, Mr. President, under all the data obtainable, I insist that the evidence is against the necessity for operative treatment by trephining with or without excision of brain substance in idiopathic epilepsy; also in Jacksonian epilepsy and in focal epilepsy. Nor must it be taken for granted that every epileptic who has a depressed fracture or a lesion of the dura or brain must of necessity be trephined. Every such case stands on its own individuality, the treatment must be adjusted to the indications of each case. As "The American Text-book of Surgery" wisely remarks: "Each case of epilepsy must be studied by itself for days, and sometimes even for weeks, before we reach a definite conclusion that it is wise to operate. The surest way to discredit cerebral surgery is to practice unwise cerebral surgery. Hence, while advocating a more radical treatment than that formerly thought to be wise, we must be careful not to proceed to the opposite extreme of indiscriminate operative interference. A progressive conservatism, if the term is allowable, should be our rule."

Now, in this spirit I insist that in advanced epilepsy, or I should say chronic epilepsy in connection with a depressed fracture, the hope of cure by trephining is very small. Why? Because the epileptic habit has already been formed, and the brain degeneration consequent upon repeated epileptic convulsions has in a great majority of cases already taken place. The same is true of epilepsy consequent upon cerebral tumors as well as those cases the result of cicatrices of the brain or the dura. If the history of the operative treatment of epilepsy proves any one thing above another, it is that the beneficial effect of the operation is greatly influenced by the time intervening the first convulsion and the operation. It is admitted by almost all text-writers that trephining a depressed cranium must be resorted to immediately that the epileptic seizure occurs, if we would benefit the epilepsy; *i. e.*, we must remove the source of irritation promptly in order to prevent degenerative brain changes, and prevent the epileptic habit from becoming established. Chronic epilepsy in association with a depressed fracture, or with a cerebral lesion, is rarely if ever relieved by operative interference. This is a well established principle in brain surgery; hence "The American Text-book of Surgery" lays down this rule: "In simple fracture of the vault, with marked depression, immediate preventive trephining should be done, even if there be no signs of encephalic mischief. Any violence sufficient to break a bone and depress it, there is good reason to believe, may have lacerated the brain substance and probably the dura, and may be followed either by speedy inflammation, or later in many cases by epilepsy, etc., if the depression remains." The same authority says: "If the fracture of the vault be simple, without marked depression and without cerebral symptoms, or if moderate cerebral symptoms have been present, but are abating, or if a fracture be only suspected, the case should be treated expectantly, but if any serious symptoms of intercranial mischief arise trephining should be done." Now, Mr. President, I dissent *in toto* from the view that trephining a depressed fracture giving no sign of encephalic mischief should be done as a preventive measure against possible future cerebral inflammation, epilepsy, etc. Perhaps mischief may follow such injuries in future, although giving no present signs of existing injury is too vage, too remote, to justify the risk of trephining. The very book from which I have just quoted says, on page 493, "but remembering also that while trephining is equivalent to incision down to the injury in any other part of the body, yet that it is always a grave operation, and never to be undertaken without earnest reflection and good reason." Now, sir, I insist that a progressive conservatism, such as the author just quoted so strenuously insists upon in another part of his article on trephining in epilepsy, can show no good reason to justify submitting a patient with a depressed fracture to an operation of such gravity as he admits trephining to be, merely to prevent possible brain mischief, which does not occur in more than one case in ten, and if it occurs is of but little greater gravity than the so-called preventive trephining. Think of the proposition, Mr. President. The author seriously advocates submitting ten patients with simple depressed fracture of the cranium, giving no sign of cerebral injury, to the grave operation of trephining for the reason that some one of these ten men may, or even will, develop brain mischief in future, such as cerebral inflammation, epilepsy, etc. Now, sir, neither progressive conservatism nor good reason suggests that ten men should be thus maimed for life in order that one of them may be shielded from a possible misfortune in future, which, if it did occur, would be but little more serious than the operation by which it is proposed to shield them from mere possible harm. If mere possible harm, may be's, is to fix the rule that trephining for depressed fracture of the vault is necessary or even justifiable in cases showing no sign of cerebral mischief, then I insist that progressive conservatism, good reason, demands that every case of simple fissure of the vault should likewise be trephined. Why? Because it is a well known fact that a simple fissure of the external table may be, and not infrequently is, found in association with an internal cranial table which is broken and extensively splintered. This splintered internal table may not give any sign of cerebral irritation until a future period, yet the danger of future cerebral mischief is almost if not quite as great in this latter case as in a fresh depressed fracture unattended by present cerebral mischief. There is scarcely a museum with an extensive collection of cranial fractures which has not several examples of fissure of the external table attended with splintering of the internal table. These cases are liable to be followed by cerebral inflammation or epilepsy. Why not trephine these cases immediately that they occur? Certainly the brain can and does accommodate itself better to the pressure of a depressed fracture than to the irritation of a splintering bone. Now, Mr. President, during a twenty-five years' practice of medicine I have encountered more than twenty-five cases of recent depressed fractures of the cranium. Some of them were but slight depressions, some marked depressions—some of them were simple, some compound. In only one of these cases, a compound depressed fracture, did I feel that trephining was necessary. In this one the epilepsy came on immediately after the head lesion was inflicted; the convulsions persisted for forty-eight hours after the injury, except when the patient was deeply impressed by narcotics. I advised trephining, but the patient declined to submit to the operation. He was troubled with occasional light attacks of convulsions for several more days. After this the wound healed, the convulsions ceased, and he had but one recurrence, which was six months after the injury. For fifteen years he has been in perfeet health. I refer to the case of Mr. ———, whom all of you know. I recall the following cases, which were seen by members of this academy. All of them are in perfect henlth, yet the depressed fractures were never trephined. (These cases were detailed, but are omitted from this paper.) I therefore feel that I can speak from experience on this subject. I must insist that preventive trephining for epilepsy should be resorted to only in very exceptional cases. But let us consider if or not extirpation of a cicatrix of the dura or of the brain is necessary. Now, sir, I must be permitted to express very grave doubt on the question. I very seriously question the wisdom of the operation. Why? When the cicatrix is excised what results? The formation of another cicatrix, this time in the line of the last excision. If the formation of a cicatrix at the seat of injury is the cause of epilepsy, we have no guarantee whatever after any operation for the cure of this malady that epilepsy will not recur. Why? Because the very surgical procedure inaugurated to cure the convulsive disease—*i. e.*, elevation of a depressed bone, removal of a projecting, even an imbedded fragment of bone, or the excision of a cicatrix, is in turn followed by a cicatrix which may in turn lead to a recurrence of the epilepsy for the relief of which the operation was done. Now as to the excision of brain centers for the cure of epilepsy due to focal disease.

From all the physiological and pathological data at hand, and they are abundant and well conceived, it would seem but rational to expect cure of these convulsive seizures if the offending lesions and centers were removed. But does experience justify the theory?

Every candid, intelligent student of this question must admit that it does not. If we inquire for the reasons of this failure of experience to justify theory, we are told that the center is rarely if ever destroyed, but that sclerosis, which is secondary in its nature, is set up, and that the irritation necessary to set up convulsive seizures resides in this secondary sclerosis of tissue surrounding or contiguous to the motor center. Be the explanation what it may, Mr. President, cold, hard facts of experience indubitably prove that even the removal of motor centers does not, except in rare instances, cure the epilepsy. It may be, as contended by Dowse, that the corresponding center in the other side of the brain vicariously discharges the function of the one removed. Never mind the explanation: the fact remains that the operation rarely, if ever, cures the convulsive seizures. Bad as is the failure of the operation, after having subjected the patient to the dangers of a capital surgical proceeding, there is a further danger attendant on this operation—*i. e.*, paralysis of the parts supplied by the removal of the center. So that in each instance you will have submitted the patient to all the dangers of a capital operation and failed to cure him, but you are liable to present him with a paralyzed arm or leg as a reward for his innocent trust in your skill as a physician. But the removal of a motor center does not invariably result in failure to cure epilepsy. What then? Does not this show that the operation is not only justifiable but even necessary? Hardly. The cures are so fractional in number, that they may be said to be well-nigh as scarce as hens' teeth. But suppose you occasionally cure an epileptic by removing the motor center of an arm or leg. What have you done? Substituted paralysis of a limb for epilepsy. What benefit have you conferred upon your confiding patient? You have merely swapped the devil for the witch. Oh, but you say, you have just said that when a discharging center is removed the corresponding one on the other side of the brain vicariously carries on the disordered functions of its slaughtered partner, and this is why epilepsy recurs in cases where brain lesions have been removed. Why can't the motor center on the opposite side of the brain function for its demised partner? Now, Mr. President, I have not said that a discharging center on the opposite side of the brain will not infrequently function for its deceased partner—I merely quoted Dowse's theory; but for the purposes of this argument I will affirm it, and will go further and admit that a motor center may carry on motor functions for its slaughtered fellow. Mark you, it may discharge this kindly office for a dead brother. But I respectfully insist, Mr. President, that a man's heart must be abundantly filled with the grace of God to enable him, in his sane moments, to have a leg center removed and trust to its motor function being vicariously supplied. 'Tis true, it is claimed that in children this paralysis is only temporary, while in the adult it may be permanent. I am, sir, at a loss to understand this discrepancy of effects in childhood and adolescence except upon the truth of the old adage that "it is hard to teach an old dog new tricks." Seriously, I am of the opinion that it should be legally declared malpractice for any surgeon to remove a brain center without having plainly informed his patient that paralysis of a part may ensue. If the patient is sane, he has a right to elect to take all the risks of the operation, and the surgeon who does the operation has at least this justification, should disaster occur: "He (the patient) paid his money and took his choice."

Mr. President, in discussing this subject we should bear in mind the fact that in doing trepanning for epilepsy we are but returning to primitive methods, and that every principle of our so-called modern brain surgery, except antisepsis and excision of brain substance, was known, advocated, and practiced one hundred and fifty years ago. Hippocrates did the operation of trephining. As far back as 1705, LaMotte did the operation for epilepsy, but, as at the present time, the result was but partially successful. In 1804, Cline, of England, did a successful operation for epilepsy, and reported it through the English medical journals. From then until now trepanning for epilepsy has ever and anon been advocated. Sometimes the operation was popular with the profession; sometimes it fell into *innocuous desuetude.* Why this chequered career of trepanning the skull? Some writers say it was abandoned because, prior to antiseptic surgery, the operation was well-nigh always fatal. Now, sir, I insist that medical history will prove that it was abandoned because it failed to cure. I do not deny that asepsis has decreased the fatality of trepanning the cranium, but if any of you believe that the operation was well-nigh always fatal prior to the advent of listerism, so-called, you are seriously out of tune with the facts. In 1636 the surgeons of the King of Navarre successfully removed almost all of both parietal bones. A king would not submit to a necessarily fatal operation. Saviard trephined one individual twenty times. Marechal did twelve successful trepannings, Desportes twelve, and Gooch thirteen. Chadborn trephined Philip, Count of Nassau, twenty-seven times, and after the count's recovery he considerately gave his surgeon a solemn certificate of proficiency in the art of trephining. The most convincing proof of the safety of trepanning in ante-antiseptic days is that furnished by Schmucker. He trephined one patient eleven times within thirty days. What was the result as to gravity of the operation? Schmucker's language is as follows: The patient was so little incommoded by the operation that he seldom ever went to bed after it was done." Thus, Mr. President, you will see that even in comparatively ancient times, no less than in this day of modern brain surgery, surgeons complacently bored holes in the heads of their innocent, confiding patients with as little concern as the oil prospectors in the oil regions of Pennsylvania bore holes in the ground, hoping to strike oil. The ante-antiseptic surgeons did the operation in the good old fashion, and found that man was in that day about as hard to kill as he is in this flood-tide of nineteenth century achievements. Mr. President, antiseptics lack much of being all that is essential to success in brain surgery. The patient, and most of all, sir, the operator, are each factors equal to, and I believe above, antiseptics or asepsis. We of to-day are treading on dangerous ground. The majority of operators act upon the theory that if trepanning, and even excision of the brain, be done antiseptically, the patient must necessarily recover. I deny it. This theory was born of ignorance of medical history, and is violating experience. "Well," says one of my friends, "you have only to look at the statistics to be convinced that antiseptics have rendered trephining and excision of brain substance a perfectly safe operation." I deny it. I call for the record. Statistics, unless properly handled, lie to such an extent as to make Baron Munchausen blush with shame. No, sir; until we know the nature and extent of head lesions, the manner of their infliction, and the time and surroundings of the operation, and even the skill of the operators, we can never tabulate cases and intelligently compare one bunch of operations with another. Mr. President, let me give you a few facts which will convince any man that factors other than antiseptic or disinfectant surgery markedly influence the results of surgical operations for head lesions. Gross's Surgery, 1872, tells us: "The operation of trephining in civil practice has been followed by different results in the hands of different surgeons. In the hospitals of Paris and Vienna the operation is nearly always fatal; in London, Dublin, Edinburgh, Glasgow, and other large cities of Great Britain the mortality, although also very high, is much less; in the United States, as nearly as can be arrived at, the mortality is about three in four."

So much, sir, for civil practice. What about military practice? Stromeyer says that in one of the battles during the Schleswig-Holstein war there were eight depressed fractures of the skull, with moderate symptoms of compression, which were left to nature, and seven out of eight of them recovered. Let Stromeyer tell what killed this eighth man. He says: "In the case which proved fatal there had been some meddling, as a number of pieces had been extracted several days after the injury. During the entire war every patient perished who was trephined."

During the Crimean war only four out of all the patients trephined by the English surgeons recovered. Fourteen with depressed gunshot fracture who had not been trephined recovered. Schrive says the French had an equally unfortunate experience. During the Sepoy mutiny in India every soldier trepanned died, while a number survived who were not subjected to the operation. Of the recoveries without operation were eight cases with fracture of both tables of the skull with depression. These eight cases were subjected to all the hardship and exposure attendant upon a retreat of twenty days' march.

During the Franco-German war trepanning was but seldom done on either side, and always with a fatal result.

The surgical history of the war of the Rebellion shows that during the late unpleasantness between the States trepanning was performed in 220 cases—the mortality being 56.6 per cent. In 454 cases the broken or depressed fragments were either removed or elevated by other means than the trephine, the mortality being only 39 per cent. I leave to other hands the privilege of explaining this discrepancy of mortality after trephining done by different peoples, and the fact that the greatest mortality fell to those nations having admittedly the greatest operators known to the world.

Now, Mr. President, trephining has its legitimate scope in the treatment of symptomatic epilepsy. That scope is an exceedingly narrow one, so far as curing this affection. In selected cases it is to be highly commended for the one purpose, if no other, that by its performance we are assured that we have removed the possible cause of persistence of the convulsive seizures. But excision of cicatrices of the brain or dura is, in my opinion, rarely necessary or even justifiable. Excision of healthy motor centers has no rational place in the treatment of epilepsy. Some operators speak of relieving brain pressure by merely removing a button from the skull, and seriously recommend the practice in epilepsy. Unless *hernia cerebri* results, or the membranes be punctured and the cerebral fluid removed, it is impossible for brain tension to be thus relieved. In my opinion, taking a button out of the patient's heel would afford as much relief from brain pressure as would the mere taking out a button from the skull. If any benefits result from such an operation the effect is purely a moral one. Making a trap-door in an epileptic's head merely for exploratory purposes is, in my opinion, wholly unjustifiable. Of all the structures or appliances known to me, the cranium least of all needs a trap-door.

Repeated borings into the cranium to hit a lesion, because the previous operations showed that the thought-to-be diseased section was healthy, and, therefore, the injury must be in another place, is unscientific and unjustifiable. The human head is neither wood, stone, nor earth, to be bored into to satisfy the morbid craving for search by inexact and unscientific surgeons.

Finally, Mr. President, trephining is only justifiable as a *dernier ressort* in symptomatic epilepsy, and has no legitimate position in the treatment of idiopathic epilepsy.

